# Contrasting Marine Microbial Communities of the Fram Strait with the First Confirmed Record of Cyanobacteria *Prochlorococcus marinus* in the Arctic Region

**DOI:** 10.3390/biology12091246

**Published:** 2023-09-17

**Authors:** Monika Mioduchowska, Joanna Pawłowska, Karol Mazanowski, Agata Weydmann-Zwolicka

**Affiliations:** 1 Department of Evolutionary Genetics and Biosystematics, Faculty of Biology, University of Gdansk, 80-308 Gdansk, Poland; 2 Laboratory of Plankton Biology, Department of Marine Biology and Biotechnology, University of Gdansk, 81-378 Gdynia, Poland; karol.mazanowski@phdstud.ug.edu.pl; 3 Department of Paleoceanography, Institute of Oceanology Polish Academy of Sciences, 81-712 Sopot, Poland; pawlowska@iopan.pl

**Keywords:** Atlantification, marine microbiome, metagenetics, next-generation sequencing, Spitsbergen, Greenland, *Prochlorococcus marinus*

## Abstract

**Simple Summary:**

Recently observed rapid climate change in the Arctic region affects the ecology of all organisms; however, little attention has been paid to the impact on microbial communities and large-scale microbial processes in the Arctic. Therefore, we analyzed the microbiome collected from the Greenland and Spitsbergen shelves, on the opposite sides of the Fram Strait, which is the main gateway of Atlantic water to the Arctic Ocean. We found that salinity was the most important factor shaping the microbial communities, which were also stratified by depth. Interestingly, for the very first time, we identified the cyanobacteria *Prochlorococcus marinus* in polar waters (75–77° N), whose distribution in oceans had been previously described only in temperate, subtropical, and tropical waters, between 40° N and 40° S. We believe that our results revolutionize the knowledge about the distribution of *P. marinus* in the oceans, which northward shift could have been connected with the process of Atlantification of the Arctic, which involves intensified transport of Atlantic water masses through the Fram Strait towards the Arctic Ocean. Considering the accelerated rate of climate warming in the Arctic, our results indicated that the microbiome community can be viewed as an amplifier of global change and that the Atlantification process is in progress.

**Abstract:**

The seawater microbiome is crucial in marine ecosystems because of its role in food chains and biogeochemical cycles; thus, we studied the composition of the pelagic marine microbiome collected in the upper 50 m on the opposite sides of Fram Strait: Spitsbergen and Greenland shelves. We found out that it differed significantly, with salinity being the main environmental variable responsible for these differences. The Spitsbergen shelf was dominated by Atlantic Waters, with a rather homogenous water column in terms of salinity and temperature down to 300 m; hence, the marine microbial community was also homogenous at all sampled depths (0, 25, 50 m). On the contrary, stations on the Greenland shelf were exposed to different water masses of both Arctic and Atlantic origin, which resulted in a more diverse microbial community there. Unexpectedly, for the very first time, we identified cyanobacterium *Prochlorococcus marinus* in Arctic waters (Spitsbergen shelf, 75–77° N). Till now, the distribution of this cyanobacteria in oceans has been described only between 40° N and 40° S. Considering the accelerated rate of climate warming in the Arctic, our results indicated that the seawater microbiome can be viewed as an amplifier of global change and that the Atlantification is in progress.

## 1. Introduction

Microbial communities consist of single-celled organisms; simple multicellular and symbiotic microorganisms, i.e., Bacteria, Archaea, and Eukarya; as well as biologically active entities, i.e., viruses and viroids. The seawater microbiome is crucial in marine ecosystems because it forms the basis of food chains and is fundamental for many biogeochemical cycles due to their biodiversity, abundance, and metabolic activity [1]. Moreover, bacterial symbionts/endosymbionts have also been widely detected in marine ecosystems. These symbiotic associations of bacteria and host species play key ecological roles in contributing to ecosystem biodiversity and stability [2]. Host-associated bacteria from various geographical regions can have different functions even when their habitat and environmental conditions are similar [3]. Therefore, it is important to understand the effects of bacterial symbionts on the host’s physiology and ecology.

Thus, over the past decades, numerous studies have been conducted to gain insight into the distribution and diversity of marine microbial communities in the polar oceans. Initially, the idea of “everything is everywhere” was proposed, i.e., that the majority of the seawater microbiome should be cosmopolitan, with low global diversity and rare endemic bacterial species [4]. Nevertheless, despite the huge population size and small body sizes of microorganisms, mixing of waters due to few geographical barriers, and the presence of waves and currents, later studies have challenged the hypothesis that the microbiome has a ubiquitous distribution; however, it seems that the key question is: how even is their distribution? The opposite results indicated that there was a large spatial variation in the microbiome, and bacterial composition (all bacteria present in the microbial community) varied at different locations, correlating with environmental factors [3]. Consequently, the second part of the axiom, “everything is everywhere but the environment selects” [5], has been confirmed.

In general, polar microbiomes have developed some potential survival strategies in cold environments, such as saccharide and lipid biosynthesis [6]. In turn, the biogeographic distribution of bacteria in the Arctic follows water mass distribution [7]. Interestingly, the microbial community of the Fram Strait differs geographically in terms of taxa and function, as the eastern part is influenced by Atlantic waters, while the western area is affected by Arctic water. Presumably, such a difference is due to environmental parameters related to the dynamics of phytoplankton blooms [8] that are associated with different nitrogen concentrations of the main water mass [9]. The density of the eastern microbial communities (bacterial and eukaryotic) and total bacterial productivity are higher, and these communities consist of bacteria responsible for the degradation of phytoplankton products. During an ice-free regime, the microbial community is dominated by diatoms and carbohydrate-degrading bacteria, while the community in the western part of the strait is characterized by the presence of heterotrophic taxa [10]. Moreover, it was observed that most of the marine bacterial communities are stratified [11], and depth has proven to be the factor that affects most differences between microbial communities present in different water layers [12]. Nevertheless, the abundance of some bacteria remained constant throughout the entire water column, probably filling various ecological niches [13]. Generally, surface microbial communities are better explored than those in the depths [7], and it seems that the prokaryotic richness of the Arctic Ocean increases from the surface to bathypelagic depths [14]. The surface marine waters are dominated by Alphaproteobacteria [15,16], although the ocean is also rich in species from the clades Bacterioidetes, Deltaproteobacteria, Actinobacteria, and Verrucomicrobiae [16]. Moreover, bacterial communities in the surface layer of different ocean regions consist of several abundant phylotypes, most of which are rare and their ecological functions are not characterized [17], and seasonal changes induce nutrient exchange between Arctic surface and deep waters [18]. In turn, the microbiome of deep sea water is more diverse [6], and the structure of the Arctic microbial community undergoes seasonal changes. For instance, Flavobacteria emerge in the microbiome in the north of Svalbard during summer, which decreases the phylogenetic diversity compared to the winter season [19]. However, some taxa are insensitive to seasonal changes in environmental factors [20,21]. 

The rapid rate of climate warming in the Arctic influences microbial processes, which can be perceived as sentinels, as well as amplifiers of global change [22]. The Fram Strait is the main gateway of Atlantic Waters (AW) to the Arctic Ocean, and its oceanographic regime of the Fram Strait is governed by two major surface water masses: cold, low-salinity Polar Water and relatively warm, saline Atlantic Water [23]. Polar Water occupies the eastern Fram Strait and flows southward as the East Greenland Current (EGC) along the entire Greenland Shelf margin [24] and transports drift ice from the central Arctic Ocean, which affects the surface water temperature and contributes to the local freshwater budget [23]. The West Spitsbergen coast is also under the influence of cold Arctic Water (ArW) that is transported from the north-eastern Barents Sea by the East Spitsbergen Current (ESC), also called the Sørkapp Current or the Spitsbergen Coastal Current [25]. Atlantic Water is transported northward through the Fram Strait along the Spitsbergen shelf edge as the West Spitsbergen Current (WSC), which is one of the major heat contributors to the Arctic Ocean [26]. The heat budget varies depending on the presence of the ice cover and the season; nevertheless, in recent years, there has been an increase in the temperature of the AW, resulting in the warming of the atmosphere and the disappearance of sea ice [27,28]. The warming of the WSC affects the Fram Strait, i.e., the western coast of Spitsbergen, which is the largest island of the Svalbard archipelago, and the north-eastern coast of Greenland, by the Return Atlantic Current (RAC), which is a branch of the WSC, that affects the biocenosis of the Northeast Greenland shelf by populating it with boreal species from the Barents Sea [29].

The Fram Strait recently experienced changes towards an increased presence of warmer and more saline water masses in a process called Atlantification or borealization [28,30]. Atlantification also covers the increased northward migration of Atlantic species [31,32]. Thus, progressive climate change is affecting the trophic webs of these polar ecosystems, changing the levels of primary and secondary production, energy flow, and predator pressure [33,34,35]. Responses of microbial communities to rapid climate changes in this region [36] and their functionality largely depend on immediate reaction to environmental changes [37]. Certain Arctic microbial ecosystems appear to be in fast decline as a result of climate change. At the same time, other microbial communities are shifting towards new regions [36].

Although microbial communities dominate the oceans in terms of diversity and abundance [1] and play a key role in the global biogeochemical cycle in the oceanic environment, research concerning the impact of the seawater microbiome on the Arctic ecosystems, as well as the overall characterization of the Arctic marine microbial communities, remains limited. Therefore, the objectives of the study were: (i) to describe the pelagic marine microbiome on the opposite sides of the Fram Strait, on the Greenland and Spitsbergen shelves; (ii) to identify potential symbiotic associations; (iii) to assess how their contrasting hydrographic properties affect microbial communities; (iv) to investigate whether large-scale climate-associated changes disturb the Arctic marine microbial composition by the presence of taxa previously restricted to warmer waters. 

## 2. Materials and Methods

### 2.1. Sampling

**Figure 1:**
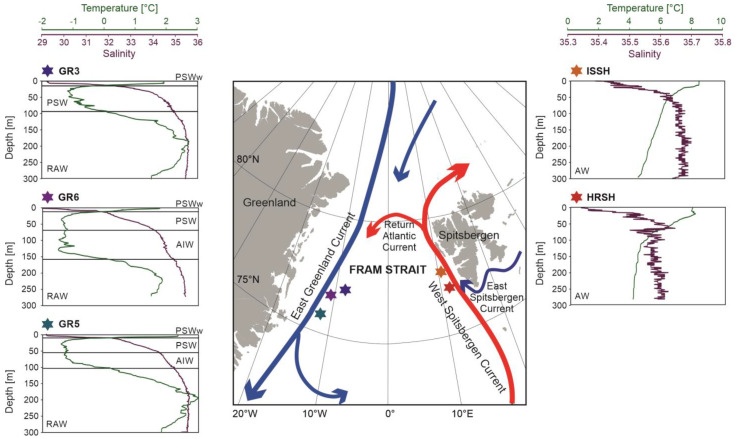
Map of Fram Strait with main ocean currents and sampling stations. Plots show changes in temperature and salinity at each sampling station and the classification of water masses based on Cottier et al. [38] and Rudels et al. [39]: Polar Surface Water warm (PSWw), Polar Surface Water (PSW), Re-circulating Atlantic Water (RAW), Arctic Intermediate Water (AIW), and Atlantic Water (AW). The map was created using ArcGIS 10.7.

Sampling was performed at 5 stations located at the shelves of western Spitsbergen and eastern Greenland during the R/V Oceania cruise in August 2018 (Table 1, Figure 1). At each station, temperature (°C) and salinity in the water column were measured using Mini CTD Sensordata SD202 (SAIV A/S, Laksevag, Norway) in 1 sec intervals.

At each station, water samples were collected from the depths of 0 m (surface), 25 m, and 50 m, chosen according to the expected values of 100% photosynthetically active radiation (PAR), maximum chlorophyll-a concentration and below, and the euphotic zone (below 1% PAR), respectively [40,41], which allowed for the collection of samples from the depths of contrasting environmental conditions, especially for the autotrophic microbial taxa. Such a sampling strategy resulted in the total collection of 9 Greenland shelf and 5 Spitsbergen shelf samples. Approximately 1000 mL of each sample was filtered subsequently through 20 µm Grade 4 and 1 µm GF/B cellulose filters (Whatman, Maidstone, UK) under low suction pressure. At first, each sample was passed through Grade 4 filter, and then filtrate was passed through GF/B filter (two filters per sample). To avoid cross-contamination, all sampling and filtration equipment were washed with 100% ethanol before the filtering of each sample. All filters were stored at −80 °C until further analysis.

### 2.2. DNA Extraction

DNA extraction from the microorganisms collected by filtration (GF/B filters) was performed using silica membranes from a commercial Genomic Mini AX Bacteria + kit (A&A Biotechnology, Gdańsk, Poland), with some modifications to the manufacturer’s instruction, in order to lyse the microbial community more efficiently. Additionally, the tubes were continuously shaken (500 RPM, Eppendorf Thermomixer compact 5350) for 3 h. Centrifugation for 20 min at 50 °C and 650 RPM was added to the first step. Moreover, a thermal shock was also added to the fourth step of the manufacturer’s instructions, i.e., the tubes were placed five times at –20 °C for 2 min, then moved to +20 °C for 2 min and centrifuged each time. All laboratory procedures were conducted with sterile equipment, and all steps were carried out in a sterile laminar flow hood to avoid cross-contamination of the samples. Extracted DNA was quantified using a NanoDrop ND-1000 UV-vis (Thermo Fisher Scientific, Waltham, MA, USA) and stored at −20 °C for further analyses.

### 2.3. 16S rRNA Amplicon Library Generation and Taxonomic Classification

The Next Generation Sequencing (NGS) of the V3-V4 hypervariable region in the bacterial 16S rRNA gene was carried out using universal bacterial primer set: V3-F: 5′-TCGTCGGCAGCGTCAGATGTGTATAAGAGACAGCCTACGGGNGGCWGCAG-3′ and V4-R: 5′-GTCTCGTGGGCTCGGAGATGTGTATAAGAGACAGGACTACHVGGGTATCTAATCC-3′ [42]. The targeted gene region was prepared applying Herculase II Fusion DNA Polymerase Nextera XT Index Kit V2 and 16S Metagenomic Sequencing Library Preparation Part # 15044223 Rev. B library protocol. Paired-end (PE; length filter (assembled read) 400 bp ≤ good sequences ≤ 500 bp) sequencing was performed with an Illumina platform (Macrogen Europe, Amsterdam, The Netherlands).

The sequenced data were processed with QIIME 1 (v1.9.0) pipeline (Quantitative Insights Into Microbial Ecology [43]). Raw paired-end read trimming (PE; 2× 301 bp); adapter sequences removal; quality control of raw reads, which were merged based on the overlaps of PE read; and error correction for areas where the two reads overlap was performed using the fastqprogram [44]. The poor-quality reads that did not meet the quality criteria (a quality score < 20 for >30% of the read length), reads that contained ambiguous base, chimeric sequence, or unpaired reads were removed [45]. After quality control, the FLASH program [46] was applied to assemble paired-end reads created by sequencing both directions of library.

Next-generation reads were clustered into operational taxonomic units (OTUs) using the cluster cut-off value of 97% using a de novo OTU picking tool of the CD-HIT-OTU program [47] based on cd-hit-est. Taxonomic classifications for PE reads were conducted using the NCBI 16S database, i.e., the main sequence of each OTU was referred to the NCBI 16S database, and taxonomic information was obtained using BLASTN (v2.9.0). The Biological Observation Matrix (BIOM) table with removed sequences classified as Archaea, mitochondria, or chloroplasts was used as the core data for downstream analyses. The results of taxonomic assignment are included in Table S1. The raw NGS reads were deposited in BioProject database under the study accession number PRJNA815464.

### 2.4. Analyses of the Marine Microbial Community

In order to check the diversity and evenness in the microbial community, α-diversity (Chao1, Shannon, and inverse Simpson indices), β-diversity (UniFrac distance), and the number of observed OTUs were estimated using normalized data sets. Alpha diversity (α-diversity) is defined as the mean diversity of species across sites or habitats on a local scale and is used to identify richness and evenness of individual taxa. In general, it expresses the distribution of species abundance in a given sample in a single number that depends on species richness and evenness. Common alpha diversity indices that measure the overall heterogeneity of a community include Chao1 Index, Shannon Index, and Inverse Simpson Index. The Chao1 index is a nonparametric estimator based on the taxon richness [48]. The Shannon diversity index is an estimator that combines richness, evenness, and taxa diversity [49,50]. In turn, the inverse Simpson index is the inverse of the classical Simpson diversity estimator of evenness, combining richness and taxa diversity, i.e., another derivative of the Simpson index [51]. While alpha diversity indices focus on community diversity within a sample, beta diversity (β-diversity; another name for sample dissimilarity) allows analysis of diversity between individual assemblages. We applied UniFrac, which is a β-diversity measure commonly used in phylogenetic analysis, as it allows us to compare effective/similarity measures in microbial communities [52].

The saturation of OTUs was also evaluated, with rarefaction curves based on the Chao1 richness estimator. All calculations were performed using the QIIME 1 (v1.9.0) pipeline. Subsequently, the weighted UniFrac distance, indicating the genetic relationship between the samples based on sample variation in the group, was used to quantify the relationship of phylogenies [52]—UPGMA tree—generated using the QIIME 1 (v1.9.0) pipeline. A comparison of bacterial community structure, i.e., a Venn diagram, was created—OTUs common to the microbiome from all stations were generated using the R 4.0.3 package. All charts created using R 4.0.3 package were performed with dplyr 1.0.2 [53], ggplot2 3.3.2 [54], tidyr 1.1.2 [53], and viridis 0.5.1 [55] packages.

To study the relationship between abiotic environmental variables (water temperature and salinity, as well as sampling layer), stations’ localization, and microbial community, constrained ordination techniques were applied in CANOCO 5 [56]. The results of a preliminary de-trended correspondence analysis (DCA), based on the length of the main gradient, pointed to a redundancy analysis (RDA) as the most appropriate analysis procedure; thus, we decided to use this analysis. The OTU abundance data were transformed [n’ = log (n + 1)] prior to further analyses, while the environmental variables were ranked, according to their quantitative importance, by manual selection based on the Monte Carlo permutation test adjusted for temporal autocorrelation [56].

### 2.5. Phylogenetical Analysis of Prochlorococcus marinus OTU

To determine the phylogenetic position of the OTU classified in our study as *Prochlorococcus marinus* (OTUdenovo12), as well as to obtain the most reliable evolutionary tree, we used 16S rRNA sequences available in GenBank, derived from the genus *Prochlorococcus*, i.e., *P. marinus* (GenBank: AF311218, CP000552) and *Prochlorococcus* sp. (GenBank: AF133834), as well as all OTUs described as *Prochlorococcus* sp. Identified by Sunagawa et al. [12] in metagenomic analysis of 243 Tara Oceans seawater samples collected across the globe (32 sequences in total, as OTUs with low frequency in the microbial community, i.e., less than 10 sequences, were removed from our analysis). As an outgroup, we applied 16S rRNA sequence of *Polaromonas aquatica* (GenBank accession number: MW024865). The aligned 16S rRNA sequences were trimmed to 404 bp. The most appropriate model of sequence evolution was determined by jModelTest 2 [57] with the assumptions of both Akaike Information Criterion (AIC) and Bayesian Inference Criterion (BIC). The evolution model GTR + G (Time Reversible model with gamma distributed rate heterogeneity) was selected. The phylogeny was tested by Bayesian Inference Criterion conducted with MrBayes v.3.2.6 [58] implemented in Geneious v.2022.0.2 software (http://www.geneious.com, accessed on 22 November 2022). The following parameters were estimated: GTR + G substitution model, including the chain length (1,100,000), heated chains (4), subsampling frequency (200), burn-in length (110,000), and heated chain temperature (0.2). Generated phylogenetic tree was edited with Inkscape 1.0 (4035a4fb49, 23 November 2022) [59]. In turn, Mega X [60] was applied to calculate the uncorrected genetic distances (p-distance) (Table S3).

## 3. Results

### 3.1. Hydrographical Conditions

Water temperature at the western Spitsbergen shelf ranged from approximately 4 °C to 8 °C and decreased towards the sea bottom (Figure 1). Surface water temperature was slightly higher at the station ISSH and reached 8.3 °C, while at the station HRSH it reached 7.9 °C. Salinity at both stations exceeded 35 in the whole water column. At the station HRSH, salinity varied between 35.3 at the surface and 35.6 at the depth of 63 m, and it dropped slightly to 35.5 at the bottom. At station ISSH, salinity ranged from 35.38 at the surface to 36.68 at the bottom. A thin layer of fresher water was noted in the upper 15 m.

Temperature and salinity in the eastern Greenland shelf were more variable than in Spitsbergen (Figure 1). Temperature varied between—1.5 °C and 3 °C, and salinity ranged from 29.2 to 35.5. At all stations, the upper 15 m was occupied by relatively warm (up to 2.5 °C) and fresh (salinity < 32) water. The decrease in temperature, followed by the increase in salinity, was noted up to ~100 m (150 m at the station GR6). The lowermost water layers were occupied by warm (up to 3 °C) and saline (salinity > 34) waters. At stations GR3 and GR5, the temperature in the near-bottom layer exceeded the temperature at the surface.

The CTD data reflected the threefold division of the Nordic Seas basin: the eastern Atlantic domain, the central Arctic domain, and the western area of AW and PW mixing [61]. Stations ISSH and HRSH were located near the main pathway of AW into the Arctic, and therefore, the entire water column was dominated by warm and saline AW. The east Greenland shelf belongs to the domain of AW and PW mixing. Water masses recorded at the Greenland shelf were noticeably colder and fresher. The uppermost water column was occupied by PW, with a thin layer of relatively fresh water that was sourced mainly from sea ice melting. The deeper layers were dominated by RAW, colder and denser than AW recorded in the West Spitsbergen Shelf.

### 3.2. General Characteristic of 16S rRNA Gene Metagenetic Library and Marine Microbiome Diversity

At least 203 OTUs, ranging from 203 to 446, were observed in the samples, which indicates that the microbial community was complex. The number of identified OTUs corresponded to sampling depth, with the lowest for the surface water samples (from 203 to 292 OTUs) and the highest for the deepest samples collected at 50 m (from 385 to 446 OTUs).

Thus, the samples were divided into three groups based on their depth: samples collected at the surface (0 m), 25 m, and 50 m. For each sample, >91,640 good-quality 16S rRNA gene sequences were obtained. Overall, 955,213 and 580,036 bacterial 16S rRNA gene sequences (average length—301 bp) were noted in the Greenland and Spitsbergen shelves, respectively (Table 2). For detailed information on the assembly result, see Table S2. The analysis of microbial communities showed that >99% of all reads were represented by sequences of Bacteria, and the remaining percentage comprised unassigned records and sequences of Archaea. Overall, this high value of reads classified as bacteria allowed for a reliable description of the number of OTUs present in the sample and a quantitative estimate of microbial community composition. The result of unsatisfactory sampling could be the loss of the rarest OTUs; however, we obtained a large number of singletons (i.e., OTUs represented by single sequences) and identified many unique OTUs (Table S1). Moreover, the alpha rarefaction graph showed that a sufficient number of reads was applied to identify OTUs. This alpha rarefaction curve flattened to the right, indicating that additional sequencing was not necessary and revealed trends suggesting that the richness of microbial communities depended on sample depths from both shelves (Figure S1) [62].

The seawater microbiome profiles in our study included a total of 12 phyla; however, all of them were identified in the Greenland shelf, while only 9 of them were observed in the Spitsbergen shelf. Pseudomonadota was the most abundant phylum in all taxonomies: from 65.82% in the Greenland shelf GR3.25m sample to 31.18% in the Spitsbergen shelf ISSH.0m sample (Table S1). Bacteroides and Cyanobacteria were the second and the third most dominant phyla in OTU classification, with abundance from 39.83% (Bacteroides; GR6.25m)/32.97% (Cyanobacteria; HRSH.0m) to 21.36% (Bacteroides; GR3.0m)/1.93% (Cyanobacteria; GR3.25m). Interestingly, no separation of Archaea by a deeper water mass was found. We identified Archaea (represented by the phylum Thaumarchaeota) in both shelves in the following samples: GR3.25m, GR3.50m, GR5.25m, GR5.50m, GR6.25m, ISSH.25m, and ISSH.50m, while no surface sample from either shelf contained Archaea in the NGS sequences (Table S1).

**Figure 2:**
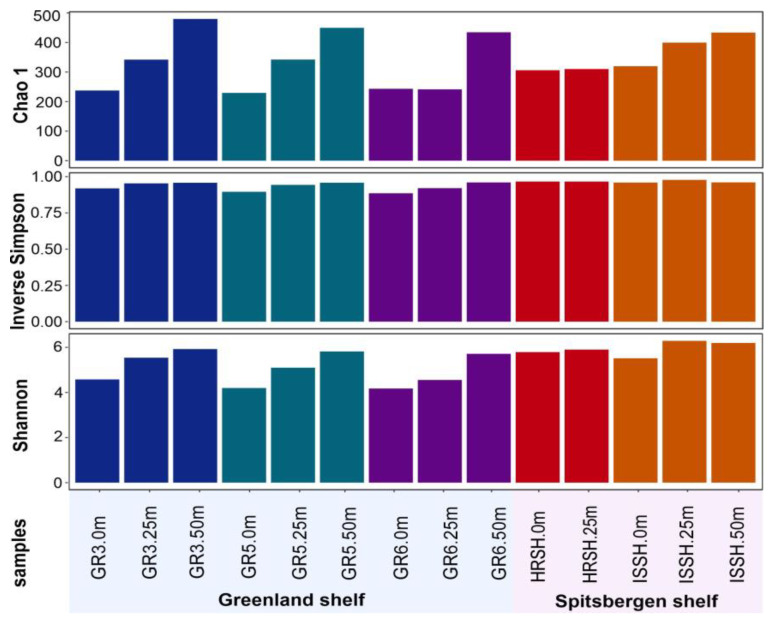
Alpha diversity indices: Chao1, Inverse Simpson, and Shannon of microbial communities present in the Greenland and Spitsbergen shelves.

Diversity indices for each marine microbial community are reported in Figure 2. Alpha-diversity analysis, which refers to the number of species in a microbial community, was performed based on rarefied 16S rRNA reads. The highest values of Chao1, Shannon, and inverse Simpson diversity indices were found for the microbial communities from the deepest samples, i.e., from the depth of 50 m. Based on these diversities, which were associated with the number of species and an even distribution of microbial species, it was demonstrated that all parameters were not dependent on the shelves but connected with depth, and the lowest values were obtained for all samples from the surface. Nevertheless, it was also shown that the samples collected from the Spitsbergen shelf were characterized by a more diverse microbial community.

### 3.3. The Influence of Hydrographic Conditions on Bacterial Communities

**Figure 3:**
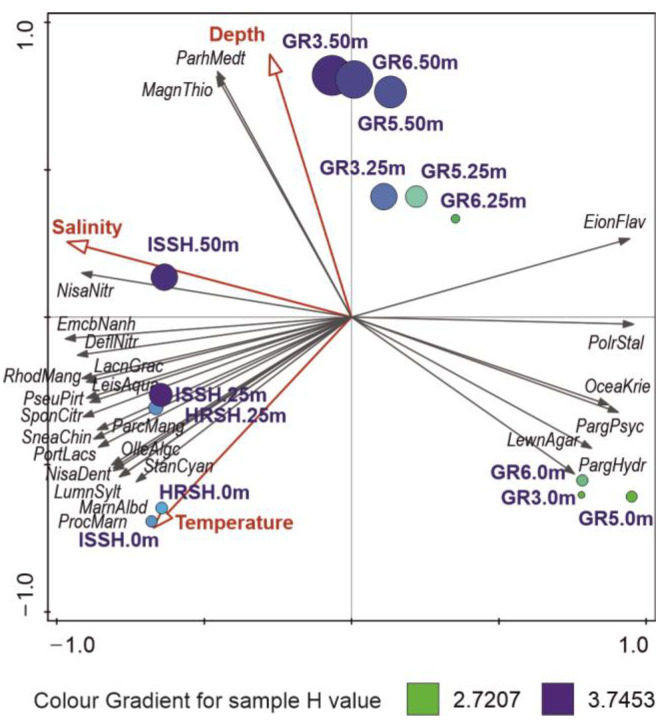
RDA ordination plot of 25 best-fitted microbial OTUs (black arrows) and their relation to significant environmental variables (red arrows). The circle size is connected to the number of OTUs, and its color corresponds to the value of Shannon diversity index H calculated for a respective sample.

According to the RDA model (F = 6.40, *p* = 0.001), salinity was the most important factor shaping the marine microbial communities of the Fram Strait. In our study, it accounted for 45.9% of the explained variability in data (Table 3), and consequently, its eigenvector was connected to the main gradient in the RDA ordination plot (Figure 3), which revealed a clear distinction between the samples from Spitsbergen shelf that were connected to higher salinity, and the surface samples from the Greenland shelf, which were characterized by the lowest salinity. The main bacterial OTUs related to the increasing salinity were *Nisaea nitritireducens*, *Emcibacter nanhaiensis*, and *Defluviimonas nitratireducens*, which were present mainly in the samples from Spitsbergen, while OTUs connected with lower salinity, and thus presenting mainly in the surface samples from Greenland, were as follows: *Polaribacter staleyi*, *Oceanobacter kriegii*, *Paraglaciecola psychrophile*, and *P. hydrolytica*.

Another factor, temperature, which is important, e.g., for enzyme activity, was responsible for 21.5% of explained variability in the studied microbial communities, and its highest values were noted in the surface samples of Spitsbergen shelf, in which the highest proportions of *Stanieria cyanosphaera*, *P. marinus* (unexpected discovery of these cyanobacteria in Arctic waters has been described in Section 3.5), *Marinifilum albidiflavum*, *Luminiphilus syltensis*, and *N. denitrificans* were observed. In turn, the sampling depth accounted in our study for 5.4% of the seawater microbiome variability, with *Magnetospira thiophila* and *Parahaliea mediterranea* being increasingly present in the deepest samples of studied both shelves (Figure 3).

Furthermore, the RDA ordination plot revealed three main groups of samples: (1) the Spitsbergen shelf, (2) surface waters of the Greenland shelf, and (3) the remaining samples from the Greenland shelf, which were connected to the above-described changes in environmental variables and dominance of different microbial OTUs and mirror the distribution of water masses in the Fram Strait (Figure 3).

**Figure 4:**
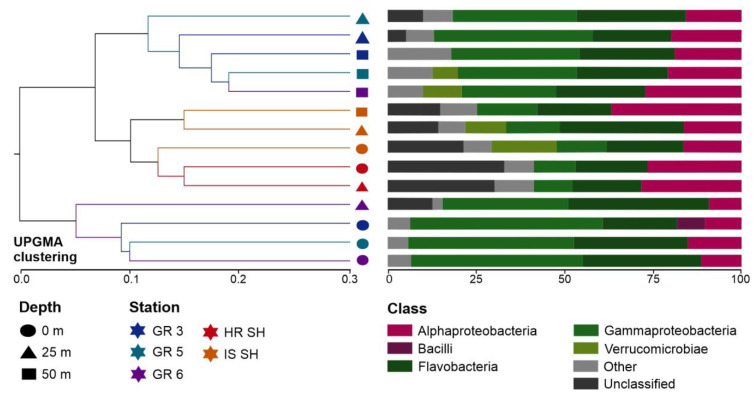
Analysis generated based on UniFrac distance matrices: UPGMA clustering of microbial communities from Greenland and Spitsbergen shelves with the abundance of bacterial 16S rRNA gene sequences at the class level.

A similar distribution of samples can be seen in the plot illustrating UPGMA sample clustering based on the UniFrac distance (Figure 4), which measures the dissimilarity between samples based on the lineages they contain [63]. Bacterial communities from the Greenland and Spitsbergen shelves were divided into two main clades. One of these distinct clades consisted of all surface samples from the Greenland shelf and one sample from this region from the 25 m depth. The second clade consisted of two subclades, i.e., one grouped all samples from the Spitsbergen shelf, and the second subclade grouped samples collected from 25 m and 50 m in the Greenland shelf region. Overall, the dissimilarity was lower between deeper samples from the Greenland shelf and all samples from the Spitsbergen shelf than the surface vs. deeper samples collected from the Greenland shelf. Flavobacteria, Alphaproteobacteria, and Gammaproteobacteria were the most abundant class of bacteria in all samples. Computed differences between microbial communities based on the phylogenetic distance between them showed that samples collected from the Spitsbergen shelf contained more unclassified sequences (Figure 4).

### 3.4. Core Marine Microbial Communities and Taxonomic Bacterial Specificity in the Greenland and Spitsbergen Shelves

**Figure 5:**
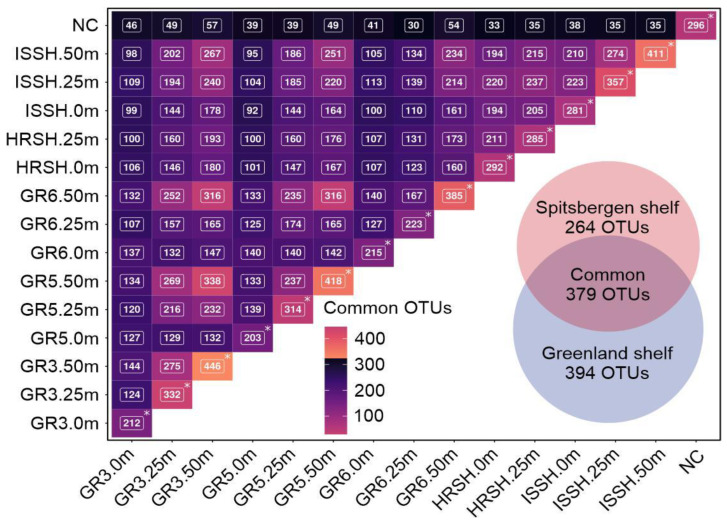
The matrix showing the number of common OTUs for the Greenland and Spitsbergen shelves with a Venn diagram illustrating the core and specific seawater microbiome OTUs of all samples. Asterisks indicate the total number of identified OTUs per sample.

The BLASTp searches demonstrated that 773 OTUs were present in the Greenland shelf samples, with 394 OTUs described as specific for this region (Polar Water), while 643 OTUs were identified in the microbiota from the Spitsbergen shelf samples, 264 of which were unique for this Atlantic Water (Figure 5). In total, 379 core seawater microbiome OTUs were common to the microbiome of both sampling regions, indicating that the microbial population was complex. The number of common OTUs shared among all samples is shown in Figure 5.

The first ten common bacterial species for the Greenland and Spitsbergen shelves were as follows: *Eionea flava*, *P. staleyi*, *Foliisarcina bertiogensis*, *Amylibacter ulvae*, *Lacinutrix venerupis*, *Sedimenticola thiotaurini*, *Porticoccus hydrocarbonoclasticus*, *Planktomarina temperata*, *Tenacibaculum aiptasiae,* and *F. bertiogensis*. The most abundant bacteria species in the Arctic Ocean, i.e., Candidatus Pelagibacter (SAR11) [64], was also found in all our samples. Nevertheless, the microbiome profiles of both shelves contained specific species of bacteria. Consequently, there were groups of bacteria species characteristic for certain regions, which are presented in Table 4. Interestingly, various cryptic taxonomic members of Deltaproteobacteria were found at all stations: (1) *Desulfohalophilus alkaliarsenatis* and *Desulfovibrio alaskensis* were recorded at 50 m depth in all GR stations; (2) *Geoalkalibacter subterraneus* was found in the ISSH station at 0 m and 25 m, as well as *Geobacter metallireducens*, which was also identified in the ISSH station, but at a depth of 50 m; (3) *Geopsychrobacter electrodiphilus* was common to all stations at 50 m (note that only surface and 25 m samples were obtained from the HRSH station).

In our study, some OTUs belonging to bacterial symbionts were identified (Table 4). For example, oxygenic photoautotroph *Acaryochloris marina* was identified on both shelves to a depth of 25 m (Table 4). In all samples from the Greenland shelf, at a depth of 25 and 50 m, *Endozoicomonas ascidiicola* symbiont was present (Table 4). We also identified the cyanobacteria *Dulcicalothrix necridiiformans*, *F. bertiogensis,* and *S. cyanosphaera* at both shelves and *P. marinus* at the shelf of Spitsbergen. Less-common cyanobacteria are shown in Table 4.

### 3.5. Unexpected Discovery of Prochlorococcus marinus Cyanobacteria in Arctic Waters

**Figure 6:**
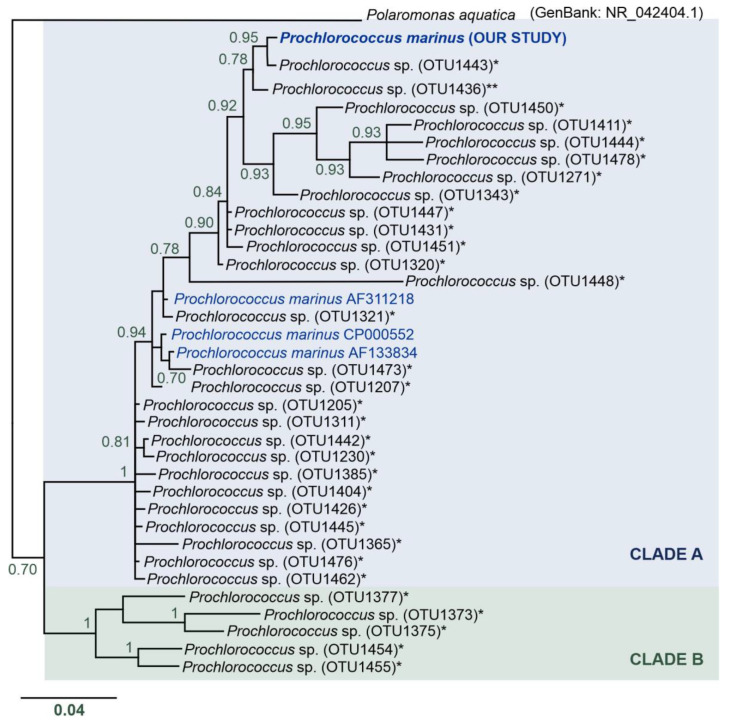
Phylogenetic reconstruction of the *Prochlorococcus marinus* identified in our study—BI analysis topology. Nodes with pp < 70 were collapsed. Clade A is indicated in blue, and Clade B in green. Asterisks (*) indicate 16S rRNA sequences of OTUs of the genus *Prochlorococcus* from the study of Sunagawa et al. [12]; double asterisk (**) indicates *Prochlorococcus* OTU found in the Antarctic Province. Sequences downloaded from GenBank are marked in blue.

Surprisingly, we found an OTU (OTUdenovo12) of *P. marinus* cyanobacterium in the Spitsbergen shelf (many sequences per station, from 283 to 4000 sequences, see Table 4, Table 5, and Table S1, Figure 6) that is ubiquitous in the mid-latitude oceans. The phylogenetic reconstruction of relationships between (i) the *P. marinus* OTUdenovo12 from our study, (ii) *Prochlorococcus* OTUs from metadata from the Tara Oceans expedition [12], (iii) and 16S rRNA sequences of *Prochlorococcus* downloaded from the public NCBI database indicated the presence of two main clades, designated by us as Clades A and B (Figure 6). The monophyly of these two clades was strongly supported (pp = 1). Clade A included very diverse and numerous *Prochlorococcus* sequences with uncorrected genetic p-distance values from 0.0 to 6.3%, with an average distance of 2.7%. In turn, Clade B consisted of a limited number of *Prochlorococcus* sequences and p-distance values ranging from 3.7 to 7.5% and an average distance of 5.5%. Overall, the divergence between the major clades was 9.5% (Table S3).

## 4. Discussion

In our study, contrasting marine microbial communities on the opposite sides of the Fram Strait were found to be connected to water mass distribution and depth stratification, with the first confirmed record of the cyanobacterium *P. marinus* in the Arctic. The surface waters of the Greenland shelf, dominated by Polar Surface Water, were characterized by the lowest number of microbial OTUs and the lowest values of Shannon diversity index H, while the deepest samples from this area showed the opposite trends. In turn, the samples collected from the Spitsbergen shelf could also be characterized by the number of OTUs and diversity increasing with depth, although without the contrasting values observed for Greenland. At the same time, samples collected from the Spitsbergen shelf, dominated by Atlantic waters with a homogenous depth stratification in terms of salinity and temperature, contained less-unique bacterial sequences. The identification of higher diversity of the microbiome community in deeper seawaters implied that they could be regarded as a reservoir of bacterial species [6]. Overall, similarly to Fadeev et al. [8], we found that the vast majority of OTUs were shared between the Fram Strait regions. Moreover, in all our samples, there was a number of unique sequences (from 236 to 2580 sequences), for which no homologous sequences have been identified in the NCBI database (Table 2). Earlier work on regional differences in microbial composition across the Fram Strait, i.e., from the West Spitsbergen Current and East Greenland Current, also reported unique OTUs for one of these regions [18].

Bacterial communities in the oceans are not homogeneous, as regional water masses with their own environmental properties, i.e., different temperatures and salinity, may carry specific bacterial assemblages. In our study, the distribution of microbial community, illustrated by the groups of samples in the RDA plot (Figure 3), mirrors the distribution of water masses in the Fram Strait: a homogenous Atlantic-dominated water column on Spitsbergen shelf; cold and less-saline surface waters on the Greenland shelf; and deeper and more-saline Polar waters. The influence of water masses of different properties on the microbiome is also confirmed by RDA, in which salinity turned out to be the most powerful environmental variable that explained the highest proportion of variation, 45.9% (Table 3). Salinity has also been previously described as a significant factor in structuring the marine microbial community in marine waters [65]. The diversity of bacterial communities in open seawaters had previously been dictated by environmental variability [66], and marine microbial community distribution in the Arctic Ocean was explained by the main factor, i.e., water masses [7,8]. Moreover, a higher similarity was found between the deep waters of the Greenland shelf and samples from the Spitsbergen waters than between the surface and deep waters of the Greenland shelf. In turn, an increase in the number of OTUs with sampling depth is consistent with a previous metagenomic study concerning seawater samples from different depths (Arctic Surface—0–100 m depths and Arctic-Deep—200–4000 m depths [6]; seas around Svalbard—1–1000 m depths [18]).

Analyzing the structure of the marine microbial community is critical to defining the role bacteria play in ecosystem processes. However, studies concerning the diversity of marine microbial communities in the Arctic region are limited, especially of pelagic communities [7,14,15]. As an example, the correlation between bacteria occurs with depth, as Actinobacteria, Bacillota, Beta-, Delta-, and Gammaproteobacteria emerge, and at the same time, Bacteroidetes and Alphaproteobacteria become in lower abundance. The classification of OTUs at the phylum level revealed the dominance of Pseudomonadota (Alphaproteobacteria and Gammaproteobacteria) and Bacteroidetes in the Arctic surface waters [6,7]. In turn, Deltaproteobacteria were most abundant in deeper waters [6,7]. The analysis of polar summer microbial communities indicated that Gammaproteobacteria and Flavobacteria were the two main bacterial taxa in the Fram Strait in both ice-free (eastern) and ice-covered (western) regions [8]. These observations contradicted the study by Cardozo-Mino et al. [13], who described the dominant abundance of Gammaproteobacteria and Bacteroidetes; however, the latter authors applied CARD-FISH and a semi-automated counting methodology, which could have had some influence on the observed differences. Quero et al. [14] showed an abundant presence of Alphaproteobacteria in microbial communities in the Arctic waters of the eastern Fram Strait. In contrast to our results, Bacteroidetes were indicated as one of the most abundant bacterial groups in the shelf waters, and their number strongly decreased with depth, but these data were based on deeper, bathypelagic samples. In general, bacterial communities from the surface were mostly composed of heterotrophic and phototrophic organisms. Deeper waters were inhabited by a microbiome consisting of mostly chemotrophic bacteria [67].

Symbiotic associations between bacteria and animals are common in marine environments and vary between genera [68]. In our study, oxygenic photoautotroph and facultative symbiont *A. marina* and symbiotic *E. ascidiicola* were identified, which were probably associated with ascidians present in the Fram Strait. *A. marina* photosymbiont was previously found in the *Lissoclinum patella* [69]. Later, the presence of *Acaryochloris*-like cells was also reported in other ascidian species [70]. Although ascidian-cyanobacterial symbioses have been proposed to be mutualistic, the role of photosymbionts in these relationships remains unknown [71]. Previously, *E. ascidiicola* was isolated from *Ascidiella* sp. and *A. scabra* [72]. Generally, *Endozoicomonas* are host-specific and facultative symbionts of marine ascidians [73].

According to previous studies, Cyanobacterial sequences were expected to be almost completely absent in the marine Arctic waters [14,74]; thus, their presence in the study area was unexpected. We especially believe that our findings revolutionize knowledge about the distribution of *Prochlorococcus* in the oceans because, for decades, it has been commonly described only in the region between 40° N and 40° S as the most abundant *Prochlorococcus* and a primary producer in subtropical and tropical waters [75]. For instance, in 2002, Bano and Hollibauhg [76] claimed that these important members of plankton communities were not found in polar oceans and were widely distributed only in temperate and tropical oceans. Similarly, Biller et al. [77] argued that *Prochlorococcus* was restricted to warmer, oligotrophic oceans and absent from colder, nutrient-rich waters at high latitudes.

The *Prochlorococcus* OTU identified in our study belongs to Clade A (Figure 6). This clade clusters *P. marinus* sequences from the GenBank database with uncorrected genetic p-distances (compared to our sequences) in the range of 2.6–2.9% (Table S3). Two *Prochlorococcus* OTUs identified by the *Tara* Ocean expedition were found to be the closest relatives of the *P. marinus* discovered in the Spitsbergen shelf. Interestingly, these OTUs were ubiquitous and had wide distribution: (i) most similar with p-distance of 0.3%—*Prochlorococcus* OTU1443—found in the microbial communities of almost all marine waters across the globe, with the exception of only two localities, i.e., South Atlantic Ocean—Southwest Atlantic Shelves Province (locality code: FKLD) and the Southern Ocean—Antarctic Province (locality code: ANTA); (ii) the second very similar sequence with p-distance of 1% was represented by a ubiquitous *Prochlorococcus* OTU1436 identified in all investigated oceanic stations, and it was a single unique OTU found in the Southern Ocean (Antarctic Province); however, the authors did not describe this record in the literature and we found these data in the available supplementary material [12]. Based on the phylogenetic reconstruction of relationships between the *P. marinus* OTUdenovo12 from our study, *Prochlorococcus* OTUs from the *Tara* Oceans expedition metadata [12], and 16S rRNA sequences of *Prochlorococcus* downloaded from the public NCBI database, we speculate that Clade A included a complex of *P. marinus* species, while Clade B could represent other species of the genus *Prochlorococcus*. Nevertheless, our molecular data are too sparse to perform species delimitation, and this interesting issue requires more in-depth analysis in the future, especially since the previous experiments indicated that the entire *Prochlorococcus* group differs by 3% at most based on 16S rRNA sequences [78].

The *Tara* Oceans expedition collected marine microbial community samples from all main oceanic regions, except for the Arctic, i.e., from the North Atlantic Ocean 44° N (North Atlantic Subtropical Gyral Province) to the Southern Ocean 62° S (Antarctic Province). The abundance of *Prochlorococcus* in the microbial communities of investigated oceanic regions was as follows: North and South Pacific Ocean from 0.07 to 13.35%, North and South Atlantic Ocean from 0.0001 to 10.98%, Mediterranean Sea from 1.51 to 10.72%, Red Sea from 2.81 to 18.34%, Indian Ocean from 0.09 to 18.66%, and Southern Ocean from 0.00006 to 0.03%. For the Antarctic region, higher values of *Prochlorococcus* abundance were determined in surface waters (5 m) compared to deep waters (90–790 m). This observation was congruent with our data obtained at both the ISSH and HRSH stations in the Spitsbergen shelf, where the abundance of *P. marinus* decreased with depth (Table 5). Moreover, West et al. [79] also indicated that *Prochlorococcus* genotypes showed significantly different depth distributions in the North Atlantic Ocean and the Red Sea. Interestingly, in the available supplementary data of the study by Cao et al. [6], we found trace amounts of *Prochlorococcus* sequences in the Arctic and Antarctic microbial communities (0.002% to 0.4%), and these low values did not correspond with sample depths. Unfortunately, the classified OTUs were not available, and we could not perform a comparison with our data.

Overall, the temperature was shown to be the main environmental parameter explaining the distribution of *Prochlorococcus* and other marine Cyanobacteria, i.e., *Synechococcus* [80]. In our study, the temperature was also correlated with the abundance of *P. marinus*, as can be seen in the RDA ordination. Likewise, the temperature has been described as the major driver of the global distribution of *Synechococcus*—a picocyanobacterium that has a high impact on ocean ecosystems, as it is responsible for c.a. 17% of ocean net primary productivity. Till (2016) [81] generally observed that *Synechococcus* was almost absent in polar oceans; however, increasing ocean temperature in high-latitude systems caused subsequent ecosystem changes, and *Synechococcus* has been documented in the Arctic Ocean north of 79° N [81]. In our study, only three *Synechococcus* sequences were identified at ISSH in surface waters; thus, its low abundance was not sufficient to discuss its possible impact on the Fram Strait region.

Nevertheless, as global climate changes, the Arctic is warming up significantly faster than other regions, and changes in water masses transport and distribution are also currently being observed there, including the process of Atlantification [28]. As the gateway of Atlantic waters into the Arctic, the Fram Strait is especially vulnerable, which results in multiple changes, including shifts in the distribution of marine planktonic species [31,82]. In light of the above, the identification of *Prochlorococcus* in polar waters should not come as a surprise, as cyanobacteria have been found to consist of distinct physiological lineages, and these differences allow proliferation under a wide range of environmental conditions [78]. However, it should be emphasized that the presence of this cyanobacteria in our samples does not prove that it is able to reproduce and grow in the Arctic, not only transported with the ocean currents. Therefore, we suggest that future research in these high-latitude environments, including *P. marinus’s* (as well as *Synechococcus*) distribution in the Arctic Ocean, is needed to support the overwhelming microbial evidence of Atlantification in the contrasting marine microbial communities of the Fram Strait, which is the main northward Atlantic waterway. Future research focusing on the microbial communities of the Fram Strait should also ideally be conducted throughout the year to gain insight into the microbiome’s seasonal dynamics.

## 5. Conclusions

1. Contrasting marine microbial communities on the opposite sides of the Fram Strait were connected to water masses distribution and depth stratification, and salinity was the most important factor shaping these communities.
2. The presence of some bacteria was restricted to one area, i.e., *Dulcicalothrix necridiiformans*, *Lewinella xylanilytica*, *Olleya marilimosa*, *Rubritalea marina,* and *Vicingus serpentipes* were found only on the Greenland shelf, while others, like *Dulcicalothrix necridiiformans*, *Leisingera aquaemixtae*, *Luteibaculum oceani*, *Marinifilum albidiflavum*, *Olleya algicola*, *Phaeocystidibacter marisrubri*, *Portibacter lacus*, *Porticoccus hydrocarbonoclasticus*, *Pseudofulvibacter gastropodicola*, *Pseudohongiella nitratireducens*, and *Roseibacillus persicicus,* were present only on the Spitsbergen shelf.
3. The surface waters of the Greenland shelf, dominated by the Polar Surface Water, were characterized by the lowest number of microbial OTUs and the lowest diversity, while the deepest samples from this area showed the opposite trends. The Spitsbergen shelf could also be characterized by the number of OTUs and diversity increasing with depth, and this area, dominated by Atlantic waters with a homogenous depth stratification in terms of salinity and temperature, contained less-unique bacterial sequences.
4. Higher similarity was found between the deep waters of the Greenland shelf and samples from the Spitsbergen waters than between the surface and deep waters of the Greenland shelf.
5. Two main bacterial symbionts were found: *Acaryochloris marina* and *Endozoicomonas ascidiicola*; the latter was absent on the Spitsbergen shelf and in the surface waters of the Greenland shelf.
6. The presence of *Prochlorococcus marinus* on the Spitsbergen shelf, for the very first time as far north as over 77° N, could have been connected with the process of Atlantification of the Arctic, which can have serious ecological consequences.

## Figures and Tables

**Table 1:** Sampling details.

Location	Station	Latitude	Longitude	Sampling Date	Depth [m]
Spitsbergen	ISSH	77°31,349′ N	10°44,314′ E	14 August 2018	980
	HRSH	76°57,348′ N	12°59,901′ E	14 August 2018	287
Greenland	GR3	76°53,604′ N	7°04,895′ W	16 August 2018	299
	GR5	75°53,488′ N	12°56,915′ W	17 August 2018	275
	GR6	76°23,954′ N	10°38,947′ W	17 August 2018	304

**Table 2:** Characterization of the 16S rRNA gene metagenetic library.

Geographical Region	Sample ID	No. of Bacterial Reads (% Passing QC)	No. of Observed OTUs (at 97% Sequence Similarity Cut-Off)	No of Sequences for Which No Homology Sequences Were Found in NCBI Database
Greenland shelf	GR3.0m	111,709 (98.23%)	212	239
	GR3.25m	97,920 (98.27%)	332	2580
	GR3.50m	106,871 (98.28%)	446	1614
	GR5.0m	100,812 (98.35%)	203	383
	GR5.25m	121,097 (98.35%)	314	1649
	GR5.50m	105,060 (98.28%)	418	1421
	GR6.0m	107,538 (98.23%)	215	622
	GR6.25m	111,056 (98.27%)	223	236
	GR6.50m	93,150 (98.38%)	385	1024
Spitsbergen shelf	HRSH.0m	135,715 (98.45%)	292	1409
	HRSH.25m	107,635 (98.31%)	285	1037
	ISSH.0m	109,873 (98.20%)	281	1522
	ISSH.25m	107,576 (98.27%)	357	1414
	ISSH.50m	119,237 (98.41%)	411	2163

**Table 3:** Environmental variables, which significantly influenced the microbial community of Fram Strait, according to RDA forward selection.

Variable	Explains %	Contribution %	Pseudo-F	P	P (adj)
Salinity	45.9	57.5	10.2	0.001	0.00333
Temperature	21.5	26.9	7.3	0.001	0.001
Depth	5.4	6.8	2	0.025	0.04167

**Table 4:** Selected bacterial OTUs identified in the sample/group of samples. Color marking of stations: (a) Greenland shelf: GR3—blue, GR5—green, GR6 —purple; (b) Spitsbergen shelf: ISSH—orange, HRSH—red.

Bacterial Species	Samples													
	GR3			GR5			GR6			ISSH			HRSH	
	0	25	50	0	25	50	0	25	50	0	25	50	0	25
Specific bacteria for geographical region														
*Dulcicalothrix necridiiformans*	+	+	-	+	+	-	+	+	-	-	-	-	-	-
*Lewinella xylanilytica*	+	-	-	+	+	-	+	+	+	-	-	-	-	-
*Olleya marilimosa*	-	+	+	-	+	+	-	-	+	-	-	-	-	-
*Rubritalea marina*	+	+	+	+	+	+	+	+	+	-	-	-	-	-
*Vicingus serpentipes*	+	+	+	+	+	+	+	-	+	-	-	-	-	-
*Dulcicalothrix necridiiformans*	-	-	-	-	-	-	-	-	-	+	+	+	+	+
*Leisingera aquaemixtae*	-	-	-	-	-	-	-	-	-	+	+	+	+	+
*Luteibaculum oceani*	-	-	-	-	-	-	-	-	-	+	+	+	+	+
*Marinifilum albidiflavum*	-	-	-	-	-	-	-	-	-	+	+	+	+	+
*Olleya algicola*	-	-	-	-	-	-	-	-	-	+	+	+	+	+
*Phaeocystidibacter marisrubri*	-	-	-	-	-	-	-	-	-	+	+	+	+	+
*Portibacter lacus*	-	-	-	-	-	-	-	-	-	+	+	+	+	+
*Porticoccus hydrocarbonoclasticus*	-	-	-	-	-	-	-	-	-	+	+	+	+	+
*Pseudofulvibacter gastropodicola*	-	-	-	-	-	-	-	-	-	+	+	+	+	+
*Pseudohongiella nitratireducens*	-	-	-	-	-	-	-	-	-	+	+	+	+	+
*Roseibacillus persicicus*	-	-	-	-	-	-	-	-	-	+	+	+	+	+
Bacterial symbiont														
*Acaryochloris marina*	+	-	-	+	+	-	+	-	-	-	-	-	+	+
*Endozoicomonas ascidiicola*	-	+	+	-	+	+	-	+	+	-	-	-	-	-
Cyanobacteria														
*Brasilonema tolantongensis*	+	-	-	+	-	-	+	-	-	-	-	-	+	-
*Chamaesiphon minutus*	-	-	-	-	-	-	-	-	-	+	-	-	-	-
*Cylindrospermum stagnale*	+	-	-	-	-	-	-	-	-	-	-	-	-	-
*Prochlorococcus marinus*	-	-	-	-	-	-	-	-	-	+	+	+	+	+
*Scytonema hofmannii*	-	-	-	-	+	-	-	+	-	-	-	-	-	-

**Table 5:** The number of identified sequences of *Prochlorococcus marinus* and its abundance in the microbial community on the Spitsbergen shelf.

Stations	Spitsbergen Shelf Station ISSH			Spitsbergen Shelf Station HRSH	
Depth	0 m	25 m	50 m	0 m	25 m
Number of identified *P. marinus* sequences	4000	829	283	2811	1826
Abundance of identified *P. marinus* in microbial communities	10%	2%	1%	6%	5%

## Data Availability

The raw NGS reads are deposited in the BioProject database under the study accession number PRJNA815464.

## Supplementary Materials

The following supporting information can be downloaded at: https://www.mdpi.com/article/10.3390/biology12091246/s1, Figure S1: Alpha rarefaction curve; Table S1: List of operation taxonomic units (OTUs); Table S2: The results of assembly; Table S3: Estimates of evolutionary divergence between 16S rRNA sequences based on p–distances. Clade A is indicated in blue, and Clade B in green. Black asterisks indicate 16S rRNA sequences of OTUs of the genus *Prochlorococcus* from the study of Sunagawa et al. [12]; pink asterisk indicates *Prochlorococcus* OTU found in the Antarctic Province. Sequences downloaded from GenBank are marked in blue.
