# Supplementary material for: Contrasting Marine Microbial Communities of the Fram Strait with the First Confirmed Record of Cyanobacteria Prochlorococcus marinus in the Arctic Region
Source: Biology (Basel). 2023 Sep 17;12(9):1246. doi: 10.3390/biology12091246 (PMC10525857; doi:10.3390/biology12091246)
Supplement: Supplementary file 1 [file biology-12-01246-s001.zip › Table S2.pdf]

Table S2. The results of assembly.

SampleName	Total Bases	Read Count	GC (%)	Q20 (%)	Q30 (%)
GR3.0m	51 263 369	111 709	51.36	98.23	94.34
GR3.25m	44 591 427	97 920	50.85	98.27	94.34
GR3.50m	48 684 583	106 871	50.93	98.28	94.48
GR5.0m	46 073 412	100 812	51.01	98.35	94.67
GR5.25m	55 109 757	121 097	50.01	98.35	94.69
GR5.50m	47 850 157	105 060	50.74	98.28	94.45
GR6.0m	49 230 172	107 538	50.85	98.23	94.35
GR6.25m	50 741 237	111 056	50.29	98.27	94.46
GR6.50m	42 327 077	93 150	50.8	98.38	94.72
HRSH.0m	60 856 955	135 715	49.8	98.45	94.92
HRSH.25m	48 296 095	107 635	49.73	98.31	94.5
ISSH.0m	49 704 925	109 873	50.38	98.2	94.2
ISSH.25m	48 800 462	107 576	50.04	98.27	94.39
ISSH.50m	53 657 208	119 237	50.2	98.41	94.84
NC	52 936 567	116 196	53.37	98.08	93.79

Total Bases : The total number of bases in reads identified.
Read Count : The total number of sequence reads.
GC(%) : The GC percentage in sequence reads.
Q20(%) : The percentage of bases in which the phred score is above 20.
Q30(%) : The percentage of bases in which the phred score is above 30.
