# Supplementary figures and images for: Contrasting Marine Microbial Communities of the Fram Strait with the First Confirmed Record of Cyanobacteria Prochlorococcus marinus in the Arctic Region
Source: Biology (Basel). 2023 Sep 17;12(9):1246. doi: 10.3390/biology12091246 (PMC10525857; doi:10.3390/biology12091246)

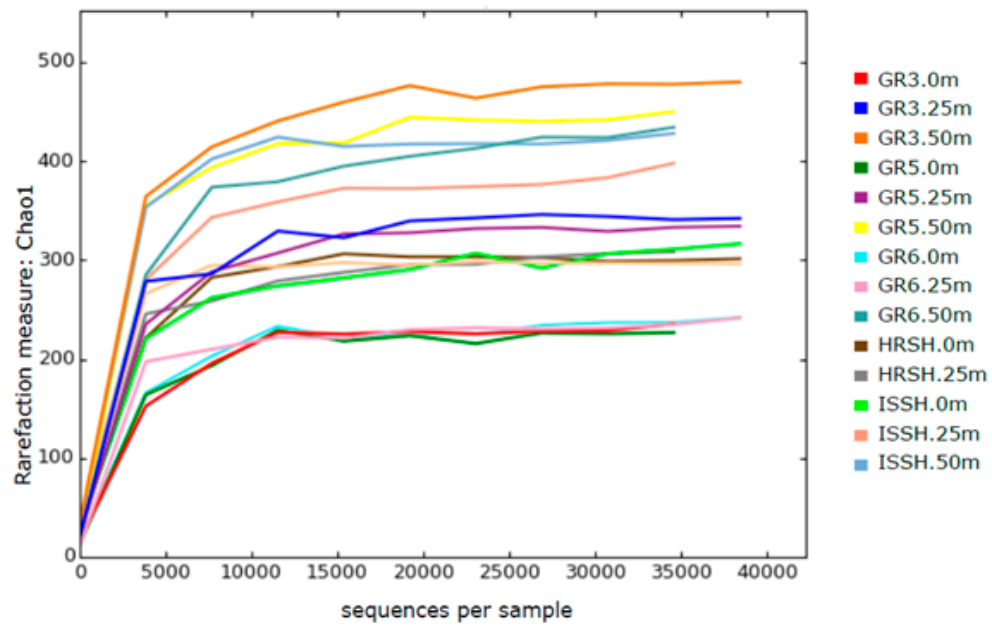

**Figure S1.** Alpha rarefaction curve.

Supplement: Supplementary file 1 [file biology-12-01246-s001.zip › Figure S1.pdf]
